# Mechanism of coordinated access to orphan drugs

**DOI:** 10.1186/1750-1172-7-S2-A24

**Published:** 2012-11-22

**Authors:** R DeRidder, C Adriaens, D Kleinermans, M Mortier, A Quanten, F Arickx

**Affiliations:** 1 NIHDI National Institute for Health and Disability Insurance (RIZIV INAMI) Belgium

## Context

Although the EU Council stated[1] that “*All health systems in the EU aim to make provision*, *which is patient-centered and responsive to individual need”*, numerous sources show important and unacceptable differences in access to orphan drugs in the Member States of the European Union (EU COM[2], EURORDIS[3,4], BE EU Presidency[5,8], EU Council[6]). With this regard, in the context of the 2010 Belgian EU presidency initiative on ‘Innovation and Solidarity’ and within the framework of the process on corporate responsibility in the field of pharmaceuticals[7], EU Commissioner Tajani launched the project Mechanism of Coordinated Access to Orphan Drugs.

## Objectives

Design a concrete operational mechanism of coordinated access to orphan drugs for patients, stakeholders and Member States. Through coordination and cooperation between stakeholders and Member States at EU level, real access is to be provided to orphan medicinal products for patients with unmet medical needs and for whom these solutions would otherwise be out of reach – in an affordable and sustainable way (“real life access”).

## Methodology

The project is managed by Belgium(NIHDI), supported by the European Commission (ENTR, SANCO, COMP, MARKET) and Eminet. Thirteen other Member States (Austria, Estonia, Finland, France, Greece, Hungary, Italy, Malta, Netherlands, Poland, Portugal , Spain, Sweden) are participating, together with the different stakeholders (AIM, EPF, ESIP, EURORDIS, CPME, EFPIA, EGA, EuropaBio, GIRP). Three Work packages (WP) cover the three different aspects of granting effective access to medicines (WP1: Identifying and assessing a relevant orphan drug , WP2: Selection of the target population and mechanisms of funding , WP3: Treatment ). In each WP operational steps and implementing activities were identified. Feasibility at present and opportunities for near future development of desirable coordinated activities were studied, and no-go solutions were documented and rejected. Integrating the three WP will lead to the development of implementable scenarios for pilot projects and result in policy recommendations.

## Discussion

Guaranteeing an added value for all stakeholders and especially from a patient’s perspective through cooperation and coordination is the main objective of the project. Although coordinated access at an European level will be organised on a voluntary basis, at some point in time, some sort of commitment from the participating partners is required. Moreover, it is crucial that the subsidiarity principle is not compromised in any way. Duplication of efforts will be avoided and previously made investments – in terms of financial and human resources, expertise and experience *-* (*ex. by EUnet HTA*, *EMA COMP*, *EUCERD*, *CAVOD*) will be valorised.
